# DART-bid for loco-regionally advanced NSCLC: Summary of acute and late toxicity with long-term follow-up; experiences with pulmonary dose constraints

**DOI:** 10.1007/s00066-016-1095-4

**Published:** 2017-01-23

**Authors:** Karl Wurstbauer, Franz Zehentmayr, Heinz Deutschmann, Karin Dagn, Ann-Katrin Exeli, Peter Kopp, Peter Porsch, Birgit Maurer, Michael Studnicka, Felix Sedlmayer

**Affiliations:** 1 Institute for research and development on Advanced Radiation Technologies (radART), Paracelsus Medical University, Müllner Hauptstraße 48, 5020 Salzburg, Austria; 2 Department of Radiotherapy and Radiation Oncology , Landeskrankenhaus, Paracelsus Medical University Clinics, Salzburg, Austria; 3 Departement of Pneumology, Paracelsus Medical University Clinics, Salzburg, Austria

**Keywords:** Non-small-cell lung cancer, Dose-Differentiated Accelerated Radiation Therapy, Pneumonitis, Chemotherapy, Pulmonary fibrosis, Nichtkleinzelliges Bronchialkarzinom, Dosisdifferenzierte akzelerierte Radiotherapie, Pneumonitis, Chemotherapie, Lungenfibrose

## Abstract

**Background:**

To report acute and late toxicity with long-term follow-up, and to describe our experiences with pulmonary dose constraints.

**Methods:**

Between 2002 and 2009, 150 patients with 155 histologically/cytologically proven non-small cell lung cancer (NSCLC; tumor stages II, IIIA, IIIB in 6, 55 and 39%, respectively) received the following median doses: primary tumors 79.2 Gy (range 72.0–90.0 Gy), lymph node metastases 59.4 Gy (54.0–73.8 Gy), nodes electively 45 Gy; with fractional doses of 1.8 Gy twice daily (bid). In all, 86% of patients received 2 cycles of chemotherapy previously.

**Results:**

Five treatment-related deaths occurred: pneumonitis, *n* = 1; progressive pulmonary fibrosis in patients with pre-existing pulmonary fibrosis, *n* = 2; haemorrhage, *n* = 2. In all, 8% of patients experienced grade 3 and 1.3% grade 4 pneumonitis; 11% showed late fibrotic alterations grade 2 in lung parenchyma. Clinically relevant acute esophagitis (grade 2 and 3) was seen in 33.3% of patients, 2 patients developed late esophageal stenosis (G3). Patients with upper lobe, middle lobe and central lower lobe tumours (*n* = 130) were treated with V20 (total lung) up to 50% and patients with peripheral lower lobe tumours (*n* = 14, basal lateral tumours excluded) up to 42%, without observing acute or late pulmonary toxicity >grade 3. Only patients with basal lateral lower lobe tumours (*n* = 5) experienced grade 4/5 pulmonary toxicity; V20 for this latter group ranged between 30 and 53%. The mean lung dose was below the QUANTEC recommendation of 20–23 Gy in all patients. The median follow-up time of all patients is 26.3 months (range 2.9–149.4) and of patients alive 80.2 months (range 63.9–149.4.). The median overall survival time of all patients is 26.3 months; the 2-, 5- and 8‑year survival rates of 54, 21 and 15%, respectively. The local tumour control rate at 2 and 5 years is 70 and 64%, the regional control rate 90 and 88%, respectively.

**Discussion and conclusion:**

Grade 4 or 5 toxicity occurred in 7/150 patients (4.7%), which can be partially avoided in the future (e.g. by excluding patients with pre-existing pulmonary fibrosis). Tolerance and oncologic outcome compare favourably to concomitant chemoradiation also in long-term follow-up.

## Zusammenfassung

**Hintergrund:**

Das Ziel ist die Analyse von akuter und chronischer Toxizität bei Langzeitnachsorge; sowie die Darstellung unserer Erfahrungen mit pulmonalen Dosisschranken.

**Methoden:**

Zwischen 2002 und 2009 wurden 150 Patienten mit 155 histologisch/zytologisch nachgewiesenem NSCLC (6 % Stadium II, 55 % IIIA, 39 % IIIB) mit folgenden Mediandosen behandelt: Primärtumor 79,2 Gy (72,0–90,0 Gy), Lymphknotenmetastasen 59,4 Gy (54,0–73,8 Gy), elektiver Lymphabfluss 45 Gy; Einzeldosis 1,8 Gy 2‑mal täglich. 86 % der Patienten erhielten zuvor 2 Zyklen Chemotherapie.

**Ergebnisse:**

Fünf Fälle von letalen Nebenwirkungen traten auf: Pneumonitis (1), progrediente pulmonaler Fibrose bei bereits prätherapeutisch bestehender pulmonaler Fibrose (2) und Hämorrhagie (2). Pneumonitiden Grad 3 und 4 wurden bei 8 % und 1,3 % der Patienten beobachtet. 11 % zeigten einen späten pulmonalen fibrotischen Umbau Grad 2. Eine klinisch relevante akute Ösophagitis (Grad 2 und 3) wurde bei 33,3 % Patienten beobachtet, 2 Patienten entwickelten eine chronische Ösophagusstenose (Grad 3). Patienten mit Tumoren in Ober-, Mittel- und zentralen Unterlappen (*n* = 130) wurden mit V20 (Gesamtlunge) bis 50 % und Patienten mit peripheren Unterlappentumoren (*n* = 14, ohne basal-laterale Tumoren) bis 42 % behandelt, ohne dass Pneumonitiden > Grad 3 aufgetreten wären. Nur Patienten mit basal-lateralen Unterlappentumoren (*n* = 5) entwickelten Pneumonitiden Grad 4/5; die V20 (Gesamtlunge) für diese Patienten lag zwischen 30 und 53 %. Die mittlere Lungendosis (MLD) lag für alle Patienten unterhalb der von QUANTEC empfohlenen Werte von 20–23 Gy. Die mediane Nachbeobachtungszeit aller Patienten betrug 26,3 Monate (Range: 2,9–149,4) und die der lebenden Patienten 82,2 Monate (Range: 63,9–149,4). Die mediane Gesamtüberlebenszeit aller Patienten betrug 26,3 Monate, die 2-, 5- und 8-Jahres-Überlebensraten lagen bei 54, 21 bzw. 15 %. Die lokale Tumorkontrollrate nach 2 und 5 Jahren betrug 70 bzw. 64 %, die regionale Kontrollrate 90 bzw. 88 %.

**Diskussion und Schlussfolgerung:**

Insgesamt 7/150 (4,7 %) Patienten zeigten eine Toxizität Grad 4/5, die in Zukunft teilweise vermieden werden kann (z. B. durch Exklusion von Patienten mit präexistenter Pulmonalfibrose). Abgesehen davon erscheinen Toleranz und klinische Ergebnisse auch im Langzeitverlauf vorteilhaft gegenüber konkomitanter Chemoradiotherapie.

## Introduction

In all, 30–35% of non-small-cell lung cancer (NSCLC) patients are initially diagnosed with locoregionally advanced disease. Radiotherapy, often in combination with chemotherapy, is the cornerstone of treatments for these patients. The ability to apply effective radiation doses is, as in all patients and all tumour sites, limited by the tolerability of normal tissues. Before volumetric arc therapy (VMAT) techniques became widely available, the method of target splitting was a decisive step towards higher dose delivery than was possible with standard three-dimensional (3D) conformal techniques [1].

We used target splitting for treating patients with nonresected NSCLC in stages II–IIIB in accelerated fractionation (termed DART-bid [dose-Differentiated Accelerated Radiation Therapy, 1.8 Gy twice daily]) and reported the results of a phase I/II trial and a consecutive prospective study with minimum follow-up times of 2 years [2, 3].

In this article, acute as well late toxicity at long-term follow-up (minimum 5 years) of all patients are critically re-assessed and summarized. In addition, our experience regarding pulmonary constraints with this novel approach is described. Figures for survival and tumour control are also updated.

## Methods

Between 2002 and 2009, 183 consecutive patients diagnosed with locally advanced lung cancer (stages II/III) were referred to our department. Patients with Pancoast tumours (*n* = 7), lack of curative possibility (*n* = 11) and potentially curable patients in very poor general condition (*n* = 15) were excluded from the present analysis (Table 1). The remaining 150 patients form the study population of this report, which comprises three subcohorts: 23 patients of a pilot study in 2002/2003 having received 84.6 Gy (mean dose) to the primary tumours and 63 Gy to nodes, respectively [2]; 123 patients, enrolled in a prospective trial between 2004 and 2009, where primary tumours were treated with increasing doses in 4 bins (73.8 Gy–90.0 Gy) depending on tumour size [3], and 4 patients treated in analogous mode in the time between these two studies (Table 1 and 2). Hence, the total cohort represents the clinical reality at a tertiary referral centre unlike patient selections frequently observed in randomized phase III trials.

**Table 1:** Overview of all referred NSCLC patients in stages II and III within the study periods

*A.*	*DART-bid doses not applicable for constraints plexus brachialis*		
	Pancoast tumours		7
*B.*	*Lack of a curative possibility*		
	Malignant pleural effusion	7	11
	Malignant pericardial effusion	1	
	Pretherapeutic fibrosis (referred after inclusion of 2 patients with pretherapeutic fibrosis in the studies)	1	
	Metastasis in the same lobe (T4)	1	
	3 Primary tumours simultaneously	1	
*C.*	*Potentially curable patients, not included in the studies*		
	Performance status strongly reduced, partially with vena cava superior syndrome	10	15
	Simultaneous extrathoracic malignancy	2	
	Bronchioloalveolar carcinoma	1	
	Insufficient dose	1	
	Refusal of the patient	1	
*D.*	*Patients included in the studies*		150

*DART-bid *dose-differentiated accelerated radiation therapy, 1.8 Gy twice daily, *NSCLC* non-small-cell lung cancer

**Table 2:** Patient (*n* = 150) and tumour (*n* = 155) characteristics

Age, years, median	65 (44–87)	
Gender: male/female, *n*	112/38	
Weight loss >5%/3 months, *n* (%)	39 (26)	
Karnofsky Index, *n* (%)	60	5 (3)
	70	60 (40)
	80–100	85 (57)
AJCC stage (6^th^ edition), *n* (%)	II	10 (6)
	III A	82 (55)
	III B	58(39)
FDG-PET staging, *n* (%)	105 (70)	
Atelectasis/dystelectasis initially, *n* (%)	47 (32)	
Histology/cytology, *n* (%)	Squamous cell carcinoma	97 (62)
	Adenocarcinoma	40 (26)
	NSC – n. o. s.	18 (12)
Gross tumour volume (ccm, range)	Mean	85 (3–492)
	Median	63 (3–492)

*NSC – n.o.s.* Non small cell – not otherwise specified, *AJCC* American Joint Commission on Cancer, *FDG-PET* 18-fluorodeoxyglucose-positron emission tomography, *ccm* cubic centimeter

### Patient and tumour characteristics

Patient and tumour characteristics are listed in Table 2. Patients were staged according to the TNM system 6^th^ edition. Of note, 26% of patients had the prognostic unfavourable feature of weight loss >5% and 43% a Karnofsky Index ≤70%. A total of 70% of patients were PET-CT staged.

### Radiotherapy

Treatment parameters are depicted in Table 3. Notably, involved nodes were treated with lower doses than primary tumours. Treatments were applied in two daily fractions of 1.8 Gy (ICRU specification) in a median of 32 days.

**Table 3:** Treatment characteristics (150 patients, 155 tumours)

Total dose (Gy)	Primary tumour (median, range)	79.2 (72.0–90.0)
	Nodes (median, range)	59.4 (54.0–73.8)
	Nodes electively^a^ (in 87% of patients)	45.0
Fractional dose (Gy)	1.8 bid	
Interval	≥10 h	
Treatment duration (days, median, range)	32 (28–43)	
Chemotherapy before radiotherapy (patients, %)	129 (86)	
	Cycles (*n*, range)	2 (1–10)

^a^To sites about 6 cm cranial to macroscopically involved nodes

*bid* twice daily

Planning CTs were performed as “slow CTs”, with patients freely breathing or as 4D-CT/average projection (internal target volume concept). A planning CT in treatment position from the apex to the bases of the lung and dose–volume parameters is available in 72 patients for whom dose–volume histograms (DVHs) were generated as usual. In 57 patients with incomplete CT datasets of the total lung extension (dating mainly before 2005), V20 was assessed by geometric approximations. In 20 patients (13%), dose–volume parameters are not available, mostly because parts of the lower lungs were not depicted. Hence, dosimetric results refer to the 72/150 (48%) of the patients with computer-based DVHs. In contouring the lungs as organs at risk, the gross tumour volume (GTV) is excluded from the lung volume. Tissue inhomogeneities were taken into account by a pencil beam algorithm. Mostly the conformal target splitting technique was used, details have been described previously [3]. In patients with computer-based V20 assessment, the median value for both lungs (volume receiving ≥20 Gy) was 32% (range 13–53%), for the ipsilateral lung 43% (range 18–69%) and for the contralateral lung 20% (range 0–39%). A dose constraint for spinal cord was set at 45 Gy and for oesophagus at 80 Gy (measured in the centre of the oesophagus at its most exposed level). KV-based IGRT was performed by matching central anatomical structures such as oesophagus, trachea, main bronchi [4].

### Medicinal agents

In 129 of 150 patients (86%), 2 cycles of chemotherapy were administered prior to radiotherapy, in general cisplatin or carboplatin containing doublets. We sought to keep the interval between chemotherapy and radiotherapy <8 days. If parts of the oesophagus were within or near the planning target volume (PTV), an antimycotic prophylaxis was given (amphotericine B lozenges, 4 times daily, during the full course of radiotherapy). In 2002, 14 patients of the phase I/II trial received a prophylactic treatment for pneumonitis by inhalative budesonide (0.4 mg twice daily by turbohaler) routinely, starting at week 3 after radiation for a 6-week duration. Similarly, 9 patients in the following period received a prophylaxis by daily oral 25 mg prednisone, at the discretion of the treating physician for patients presumably being at risk for pneumonitis. With increasing experience, this practice was changed towards prescription of steroid therapy only if patients showed clinical symptoms or radiologic signs.

### Follow-up procedures

Patients were seen for assessment of toxicity and tumour control 6 and 12 weeks after the end of radiotherapy, then every 3 months for the first year, every 4 months during the second and third year, and every 6 months thereafter. At the first follow-up a chest X‑ray, at all other controls thoracic CT scans were performed. Local or regional tumour progression was diagnosed if there was an increase in tumour volume compared with the previous CT scan. In case of doubt, a FDG-PET CT was performed. Acute and late toxicity was scored according to the RTOG/EORTC criteria except for pulmonary toxicity grade 1, because the criterion “mild symptoms of dry cough or dyspnoea on exertion” is common in these pulmonarily compromised patients. Pulmonary toxicity is considered late if persistent or developed beyond 6 months after the completion of radiotherapy. For the purpose of this report, the records, treatment plans and all pre- and posttherapeutic radiologic material (CT and PET-CT scans, thoracic X rays) of all patients were examined. Based on our experience with high-dose accelerated radiotherapy accumulated in 15 years, we critically re-assessed initial toxicity scorings and corrected these in some cases.

### Statistical analysis

Overall survival and local tumour control rates were calculated using the Kaplan–Meier method. All time intervals refer to the start of therapy. One patient was lost to follow-up for emigration, but was disease-free at 26.4 months and censored at that point of time.

## Results

Results of 150 patients treated for 155 histologically/cytologically proven tumours are reported. The median follow-up time for all patients is 26.3 months (range 2.9–149.4 months), for survivors 80.2 months (range 63.9–149.4 months). At the time of this analysis, 20 patients are alive (19 of them disease-free, one with bone metastases); 49/31/12 patients were observed >3/>5/>8 years, respectively.

### Toxicity

Acute and chronic toxicity for lung, oesophagus and vessels are summarized in Table 4.

**Table 4:** Acute (*A*) and late (*B*) nonhaematologic toxicity according to EORTC/RTOG criteria, *n* (%)

		Grade 1	Grade 2	Grade 3	Grade 4	Grade 5
A	Oesophagus	n.a.	31(20.7)	19 (12.8)	–	–
	Pneumonitis	n.a.	3 (2)	12 (8)	2 (1.3)	1 (0.7)
	Progression of pre-existing fibrosis	–	–	–	–	2 (1.3)
B	Oesophagus	–	–	2 (1.3)	–	–
	Lung	n.a.	16 (11)	–	–	–
	Haemorrhage	–	–	–	–	2 (1.3)

*EORTC/RTOG* European Organisation for Research and Treatment of Cancer/Radiation Therapy Oncology Group., *n.a.* not assessed

### Oesophagus

A clinically relevant acute oesophagitis (i. e. > grade 1) was seen in 50/150 (33.3%) patients: 31/150 (20.7%) grade 2 and 19/150 (12.8%) grade 3, respectively. In all of these patients, the symptoms ceased within 3 months. These figures are in line with a previous report on a subset of these patients [5]. Two patients developed late oesophageal toxicity grade 3 (stenosis), both received a stent 7 and 10 months after the end of radiotherapy, respectively.

### Pneumonitis

An acute pneumonitis grade 2, 3, 4 and 5 occurred in 3, 12, 2 and 1 patient, respectively, corresponding to 2, 8, 1.3 and 0.7% of all cases.

The primary tumours of 12 patients scored grade 3 were situated in the upper, middle and lower lobes in 4, 1 and 7 patients, respectively. Ten of them showed clinical symptoms grade 1 or 2 only; however since they were treated by steroids, they have been scored grade 3 by definition of the RTOG-EORTC system (“steroids may be required”). Only one patient showed in fact clinical grade 3 symptoms (dyspnoea at rest), which was possibly also attributable to a reactivated tuberculosis and/or a local recurrence. Another patient was supported by oxygen before the treatment, improved by radiation first but returned to oxygen support after a local posttherapeutic reaction. Of note, the outcome of patients scored G3 (*n* = 12) was not compromised in terms of survival: median survival 38.3 months (range 11.9–112.6 months) versus 26.3 months of the whole cohort. Four of these patients are alive at a median time of 77.4 months (range 68.6–112.6 months).

**Fig. 1:**
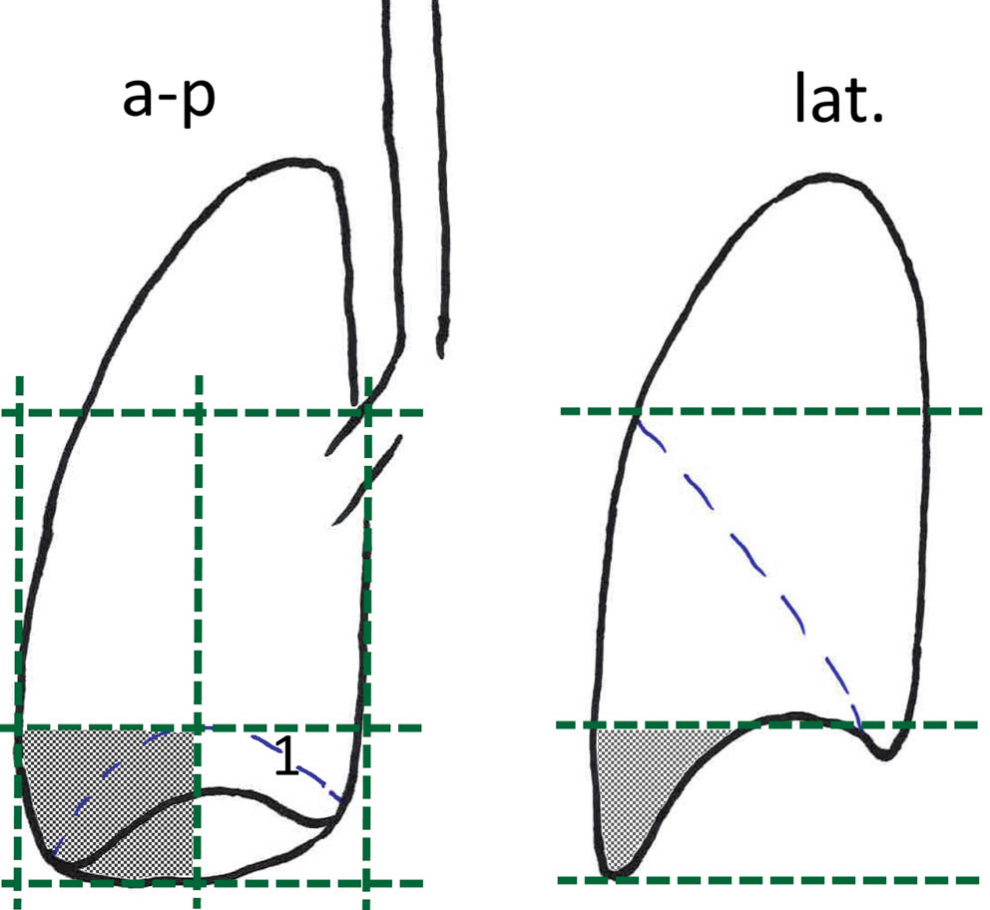
The figure shows the subdivision of the peripheral lower lobes into four parts; *1* = diaphragm; *shaded*: basal lateral part, which was the primary tumour site in patients with pneumonitis grades 4 and 5. *a-p* anteroposterior, *lat.* lateral

In 2 patients, a grade 4 and in 1 patient a grade 5 pneumonitis occurred. The primary tumours of these 3 patients were exclusively located in the basal, lateral parts of the lower lobes (Fig. 1). The grade 5 patient had a 4 cm tumour and extended mediastinal and hilar nodes; the V20 of his treatment was 53%. Six weeks post-RT the patient died with bilateral pulmonary infiltrates. One patient scored G4, also with extended nodal involvement, received a V20 of 51%; 10 weeks posttherapy a pneumonitic infiltrate first improved with steroids but returned 2 months later. Six weeks later she died due to a central, bilateral pulmonal embolism in combination with hepatic, pleural and pulmonary metastases. Another patient scored grade 4 was affected by a 2.5 cm primary tumour and subcarineal and hilar nodes only, but presented in reduced performance status. Therefore, elective nodal irradiation was not performed, and the resulting V20 was 30%. Seven weeks post-RT an infiltrate of the ipsilateral lower lobe occurred and signs of cardiac failure; the patient died within 6 days.

Regarding late toxicity, almost all patients showed local radiologic alterations of the lung parenchyma, which were scored grade 2 in 11% of the patients. Usually after 9–12 months (at the latest after 18 months), fibrotic transformations in all patients were consolidated, and no further alterations were observed thereafter.

### Pre-existing pulmonary fibrosis

Two patients with pre-existing pulmonary fibrosis died for a grade 5 toxicity: progressive pulmonary fibrosis 5 and 6 months after the end of radiotherapy and were therefore not classified as pneumonitis. These patients were treated with high pulmonary doses, which did however not exceed the usual institutional ranges (V20 of 43 and 37%, respectively). Henceforward, and as a consequence, patients with pulmonary fibrosis were excluded from high-dose DART-bid treatments.

### Pulmonary haemorrhages

In 2 patients scored grade 5, a treatment-related cause cannot be excluded. One patient, treated with 79.2 Gy for a central tumour of 4 cm with close proximity to the bronchial arteries died 6.5 months after finishing radiotherapy. The autopsy demonstrated a leakage of a bronchial artery without detection of recurrent tumour nor necrotic tissue. Another patient was treated with 88.2 Gy for a 9 cm tumour of the peripheral upper lobe with a 6 cm central necrotic cavity. The patient died due to a massive haemorrhage 4 months after therapy, possibly also caused by a cavity progression; however, since an autopsy was not performed, radiation damage cannot be excluded. In 2 further patients recurring central tumours were diagnosed prior to lethal haemorrhage; these were not classified as toxic events.

### Central tumours

A total of 21 patients with central tumours were treated with doses ≥82.8 Gy (median 84.6 Gy, range 82.8–87.3 Gy); main bronchi, lobar bronchi and sometimes also parts of the trachea lay completely or partially in the high-dose volume. Usually some narrowing of the bronchial lumen can be seen, which in some cases lead to segmental or lobar atelectasis, but alterations as cartilaginous necrosis or similar were not observed (and also no lethal haemorrhages in these patients).

### Secondary malignancies

During the follow-up period, in 11 patients twelve second lung cancers were diagnosed at a median of 24 months (range 10–62 months); four of them in the contralateral lung, five in another lobe, three in the same lobe but clearly distant from the initial primary lesion. NSCLC, SCLC and PET positive nodules without morphologic confirmation were found in 3, 2 and 7 tumours, respectively. Eleven tumours were treated with another course of radiotherapy in curative intent. The cumulative incidence of second lung cancers at 8 years is 19%. In the thoracic region no secondary malignancies other than lung tumours were observed.

In 8 patients extrathoracic secondary cancers occurred at 44 months (median; range 9–71 months) with primaries in the head and neck region (*n* = 4), colon (*n* = 2), kidney and urinary bladder (one patient each).

### Experience regarding pulmonary dose constraints

Patients with upper lobe, middle lobe and central lower lobe tumours (*n* = 130) were treated up to a bilateral V20 of 50%. For the 14 patients with peripheral lower lobe tumours, basal lateral ones excluded, V20 amounted to 42%. Despite this frequent exceeding of V20 constraints defined by QUANTEC (V20 ≤ 30–35%) [6], no cases of severe pneumonitis were observed. As for mean lung dose (MLD), the median was 16.3 Gy (range 8.6–22.3 Gy); 88% of the patients received MLDs <20 Gy, while 12% reached values between 20 and 23 Gy. Notably, and in contrast to the V20 figures, no patient transgressed the QUANTEC recommendation of MLD <20–23 Gy.

With regard to basal lateral lower lobe tumours however, 3 patients who received V20s of 53, 51 and 30% respectively, experienced grade 4/5 pneumonitis as described, whereas 2 patients with V20s of 40 and 33%, respectively, did not.

### Survival, locoregional tumour control

The median overall survival time of all patients is 26.3months; the 2‑, 5‑ and 8‑year survival rates were 54, 21 and 15%, respectively. The 5‑year survival rate for patients in stage II, IIIA and IIIB is 22%, 28% and 12%, respectively. We observed 40 local recurrences, in 90% within 2 years, resulting in a local tumour control rate at 2 and 5 years of 70 and 64%. In all, 13 patients showed an isolated regional failure, corresponding to a regional control rate of 90 and 88% at 2 and 5 years, respectively.

## Discussion

This is a summary report on acute and late toxicity of 150 widely unselected NSCLC patients in stages II–IIIB with long-term follow-up.

### Oesophagus

Compared to concurrent chemoradiation regimens, where acute esophagitis ≥ grade 2 is described in up to 44% [7], the incidence rate in our patient cohort is slightly lower (33.3%). In fact, the maximum dose constraint of 80 Gy measured in the centre of the oesophagus at its most exposed level was transgressed only by 2 patients, and only 4 patients had a mean oesophageal dose above the 34 Gy recommended by QUANTEC [8].

### Topography of peripheral lower lobe tumours

As grade 4/5 pneumonitis arose only in patients with peripheral lower lobe tumors, the topography of these tumors was studied more intensely. The lower lobes, as presented in the CT scans (lung window) of the PET-CT scans, were subdivided into four parts: basal lateral, basal medial, nonbasal lateral, nonbasal medial (Fig. 1). A primary tumour was classified as “basal” if it was completely or partially depicted caudally of the apex of the diaphragm, and it was classified “lateral” or “medial” if the centre of the tumour was located within the lateral or medial half of the lobe. A total of 19 peripheral (and 18 central) lower lobe tumours were treated, distributed within the four above mentioned areas in 5, 5, 4 and 5 cases, respectively.

### Evolvement of pulmonary dose constraints

The result of three of 150 (2%) patients with grade 4/5 pneumonitis and 12/150 (8%) patients with grade 3 pneumonitis and advantageous outcome as described seems to be a reasonable result. For modern chemoradiation schedules, pulmonary toxicities grade ≥3 up to 10% are reported [7, 9–11]. According to the RTOG 0617 protocol V20 total lung was <37%. The other aforementioned studies do not explicitly state pulmonary dose constraints. In a pooled analysis of 800 patients, fatal pneumonitis occurred in 1.9% [12].

Initially in the 1990s, we respected the constraints for 3D conformal therapies prevailing at that period: bilateral V25 30% and V20 (single lung) 50% [13–15]. As we observed that treatments were well tolerated and did not even cause low-grade toxicity, we successively – if necessary – surpassed the initial level of the constraints in about a third of the total patient cohort. In the present analysis patients with upper, middle and central lower lobe tumors were treated with V20s up to 50% and peripheral lower lobe tumours (basal lateral ones excluded) up to 42%.

A precondition to keep toxicity low is the adherence to the following “rules”: apart from sequential chemoradiotherapy and tight PTV margins, we believe that a key issue lies in the minimization of dose to the ipsilateral lung and accordingly appropriate beam arrangements, also at the cost of the uninvolved contralateral lung. Constraints are frequently driven by the level of e. g. bilateral V20, *causing symptomatic pneumonitis.* In “contralateral lung dose sparing” approaches (as usually advocated), pneumonitis will arise at a relatively low target dose although V20 total lung is kept low. In contrast, “ipsilateral lung dose sparing” approaches (thus also “allowing” irradiation of the contralateral lung) will result in a higher target dose and most probably also in a higher V20 total lung as the putatively constraint determining value. Thus, a constraint is not an absolute figure, which must not be surpassed, but also depends on the technical approach. Interestingly, the MLDs of our patients were within the limits of 20–23 Gy recommended by QUANTEC [6].

The considerations of the QUANTEC report are based on conformal 3D planned therapies with conventional fractionation [6]. It proposes to limit V20 to ≤30–35% (MLD ≤20–23 Gy) in order to limit the risk of symptomatic pneumonitis to ≤20%. It states that the significance of dosimetric parameters is technique dependent and that many non-DVH-based factors, such as performance status, age and comorbidities, may affect the risk of pneumonitis. This is in line with our own experience.

On the other hand, respecting published constraints is no guarantee to be “on the safe side”. Apart from the above mentioned aspects, tumour topography seems to play a relevant role: The fact that the primary tumours of our grade 4/5 patients were located exclusively in the basal lateral part of the lower lobes is not entirely surprising. Patients with this lung cancer topography (plus N2/3 nodes) are at risk for extraordinarily high radiation exposure because great parts of the posterior sulcus and the lower parts of the lower lobe are irradiated. Applying classical constraints might be too high in these lung regions.

Interestingly, the 2 patients with basal lateral lower lobe tumours without pneumonitis presented with chronic obstructive pulmonary disease (COPD) grade 3 and 4; and the patient with COPD grade 4 is still alive and disease-free after 7.5 years. Seemingly high-grade COPD is not an obstacle for high-dose radiation treatment, at least if the tumour load is not too extended. From surgical literature it is well established that COPD patients may experience improvement of pulmonary function after thoracic surgery because of amelioration of respiratory mechanics by removal of nonfunctional tissue [16]. Thus, high-dose irradiation could have an effect similar to surgical lung volume reduction.

On the other hand, the risk of radiation induced lung injury may depend on tumour location. As stated e. g. in a meta-analysis by Vogelius mid or inferior lung tumours are associated with a higher risk of radiation pneumonitis [17]. This may reflect higher mechanical stress in the alveoli of the mid and lower lobes compared to other parts of the lungs. A different explanation could be a relatively higher proportion of irradiated normal lung tissue due to increased organ motion in the lower parts of the lung.

### Late sequelae

In almost all patients local fibrotic transformations of the lung parenchyma can be observed which however only in 11% reached dimensions to be scored grade 2. Notably, in central tumours, hilar structures could be treated with median doses of 85 Gy in two daily fractions of 1.8 Gy without causing essential toxicity. There is no evidence of malignancy induced by radiation. No thoracic second tumours other than secondary lung cancers occurred. The cumulative incidence of secondary lung cancers at 8 years in our cohort is 19%, which is in line with the reported 2–3% annual risk following surgery of lung cancer [18, 19].

### Comparison to results of other approaches

At present concurrent chemoradiotherapy is regarded as the best treatment option for patients concerned. However, for reasons of toxicity this modality is only amenable for about 30% of the patients [11]. In addition, the possibilities to improve results by dose escalation seem to be strongly compromised by the negative outcome of RTOG 0617, showing worse results applying 74 Gy instead of 60 Gy simultaneously to chemotherapy [7]. It seems that concurrent chemotherapy hampers the full potential of more intense radiotherapy; this drawback is overcome by the sequential modality of DART-bid.

Median overall survival in concurrent therapies is reported between 17 and 28.7 months [7, 11, 20]. The good result of 28.7 months in RTOG 0617 might be influenced by the strict exclusion criteria regarding comorbidities [7].

As discussed in a recently published article, assessment and reporting of local tumour control is complex; concurrent chemoradiotherapies were reported to achieve definitive local tumour control rates of up to 65% at 2 years in positively selected patient populations [7]. In a widely unselected clinically representative patient population we reached a median overall survival of 26.3 months and local and regional tumour control rates of 70 and 90% at 2 years, respectively.

### Limitations

Doses and dose–volume relationships in our patients were calculated with pencil beam algorithm. Nowadays mostly type B algorithms (e. g. collapsed cone) are used, which compute doses more accurately, especially in sites with heterogeneous tissues as in the thorax [21]. In a subset of 19 patients in our cohort the relative median decrease in V20 was 4% between pencil beam and collapsed cone. This means that a V20 of 30% (pencil beam) corresponds to 28.8% (collapsed cone). As of 2010, collapsed cone was used at our institution without changing prescription doses. Although we did not assess this systematically, differences in treatment tolerability were not observed.

It is a general weakness of the current study that for only half of the patients computer-based dose–volume histogram calculations are available. Therefore, reported figures concerning pulmonary constraints only refer to these patients.

## Conclusion

With the concept of high-dose DART-bid excellent locoregional tumour control rates are achievable, while producing acceptable toxicity also in long-term follow-up. This is primarily due to the following aspects:

- Ipsilateral lung radiation exposure is not maximally exhausted, at the cost of contralateral exposure. This is however better tolerated than usually expected with the benefit of higher target doses and subsequently high local control rates. In this respect, the target splitting treatment concept anticipated dose distribution models becoming more and more routine in times of intensity-modulated radiotherapy.
- Compared to traditional target concepts, relevant parts of the ipsilateral lung remain spared due to tight margins. In our approach, higher doses are only administered to small volumes.
- Nodal metastases are treated with lower doses than primary tumours.
- Chemotherapy is administered in a sequential instead of a concurrent mode. Thus, and unlike many concurrent chemoradiation schemes, this approach is feasible for the vast majority of lung cancer patients with locoregionally advanced stages.
